# Seco-Duocarmycin SA in Aggressive Glioblastoma Cell Lines

**DOI:** 10.3390/ijms26062766

**Published:** 2025-03-19

**Authors:** Ann Morcos, Yeonkyu Jung, Ryan N. Fuller, Antonella Bertucci, Amy Nguyen, Quanqing Zhang, Tobias Emge, Kristopher E. Boyle, Nathan R. Wall, Marcelo Vazquez

**Affiliations:** 1 Department of Radiation Medicine, James M. Slater, MD Proton Treatment & Research Center, Loma Linda University Health, Loma Linda, CA 92350, USA; amorcos@students.llu.edu (A.M.); yeonkyu.jung0338@coyote.csusb.edu (Y.J.); antonella.bertucci@cnl.ca (A.B.); tobiasemge@gmail.com (T.E.); 2 Division of Biochemistry, Department of Basic Science, School of Medicine, Loma Linda University, Loma Linda, CA 92350, USA; 3 Department of Biological Sciences, California Baptist University, Riverside, CA 92504, USA; ryfuller@calbaptist.edu; 4 Nuclear Response & Analysis, Canadian Nuclear Laboratories, Chalk River, ON K0J 1J0, Canada; 5 Proteomics Core, Institute for Integrative Genome Biology, University of California, Riverside, CA 92521, USA; amy.nguyen010@email.ucr.edu (A.N.); quanqinz@ucr.edu (Q.Z.); 6 School of Pharmacy, Loma Linda University, Loma Linda, CA 92399, USA; kboyle@llu.edu; 7 Radiobiology & Health, Canadian Nuclear Laboratories, Chalk River, ON K0J 1J0, Canada

**Keywords:** glioblastoma multiforme, duocarmycin, seco-DSA, potency, resistance, cellular mechanisms, proof of concept

## Abstract

Glioblastoma multiforme (GBM) is among the most lethal primary brain tumors and is characterized by significant cellular heterogeneity and resistance to conventional therapies. This study investigates the efficacy of seco-duocarmycin SA (seco-DSA), a novel DNA alkylating agent. Initial investigations using a colony formation assay revealed that seco-DSA exhibits remarkable potential with IC_50_ values lower than its natural DSA counterpart. Cell viability assay indicated that LN18 cells showed a markedly greater sensitivity to DSA than T98G cells. Furthermore, seco-DSA achieved its full cytotoxic effect within 8 h of drug incubation in GBM cell lines. Although seco-DSA induced a concentration-dependent increase in apoptotic cell death, the extent of apoptosis did not fully account for the observed decrease in cell viability. Instead, seco-DSA treatment resulted in significant cell cycle arrest in S and G2/M phases. These findings suggest that seco-DSA’s cytotoxicity in GBM cells is primarily due to its ability to disrupt cell cycle progression, though the precise mechanisms of action remain to be fully established, and further research is needed. Proteomic analysis of treated cells also indicates dysregulation of proteins involved in senescence, apoptosis, and DNA repair, alluding to seco-DSA-induced arrest as a major mechanism of GBM disruption. Data are available via ProteomeXchange with the dataset identifier “PXD061023”. Our reports promote the future exploration of seco-DSA’s therapeutic potential, representing a critical step toward developing a more targeted and effective treatment for GBM.

## 1. Introduction

Glioblastoma multiforme (GBM) is one of the most aggressive brain cancers, characterized by significant heterogeneity of various cell types, including astrocytes, oligodendrocytes, vascular cells, oligodendrocyte progenitor cells, neurons, immune cells, and neoplastic cells [1,2]. This diversity within tumors complicates treatment, as different cellular populations may respond variably to therapies, making single treatments ineffective [3,4]. As a result, combined therapies targeting multiple cellular modalities are emerging as a promising strategy.

There is little progress in finding effective therapies in treating GBM despite recent efforts. Standard treatment typically involves surgery followed by radiotherapy with adjuvant chemotherapy [5,6], but these approaches often result in adverse side effects and eventual therapy resistance [7]. Additionally, while surgical resection positively impacts survival, it rarely removes all tumor cells due to GBM’s invasive nature [8,9]. Therefore, safer and more effective targeted therapies and combination therapies are needed to slow disease progression and improve overall survival with fewer secondary effects resulting from radiation and chemotherapy. Current treatments include alkylating agents like temozolomide [5], which often fail to halt disease progression effectively, contributing to a low five-year survival rate (5–10%) [9,10,11]. This highlights the urgent need for safer and more targeted therapies to improve outcomes.

The duocarmycin class of antitumor antibiotics, including DSA and its in situ precursor seco-DSA, represents a potent category of DNA alkylating agents [12]. These agents selectively alkylate adenine residues at the N3 position in AT-rich regions of DNA, demonstrating high cytotoxicity at picomolar concentrations [13,14,15,16,17]. Duocarmycin SA (DSA) and seco-Duocarmycin SA (seco-DSA) stand out as the most potent members of the duocarmycin family [16]. Seco-DSA serves as a precursor of DSA and exhibits similar alkylating selectivity to its natural counterpart [17]. Notably, there have been no studies investigating the biological effects of seco-DSA in cancer cells to date. The clinical use of duocarmycins has been limited due to severe hepatotoxicity and myelotoxicity [18]. To overcome these issues, antibody–drug conjugates (ADCs) have been developed, utilizing duocarmycin as cytotoxic payloads [19,20].

A major challenge in GBM treatment is delivering therapeutic agents across the blood–brain barrier (BBB), which greatly limits drug effectiveness. The ability of seco-DSA to cross the BBB remains unclear, as no direct studies have evaluated its permeability. However, DSA’s polarity and molecular size could limit its passage across the BBB [14]. Current research on duocarmycin-based cancer therapies focuses on ADCs [12]. ADCs consist of monoclonal antibodies that target cancer-specific antigens and deliver potent cytotoxic payloads directly to cancer cells. Given its potency, seco-DSA is a promising candidate for ADC integration. At least 15 duocarmycin-based ADCs have entered clinical trials, rekindling interest in this class of drug as targeted cancer therapies. Combining ADC technology with BBB disruption strategies could enhance drug delivery to GBM tumors and improve therapeutic outcomes [21]. Another strategy involves nanoparticle-based drug delivery systems, such as exosome-loaded seco-DSA. Studies suggest that exosomes can cross the BBB, making them a potential vehicle for seco-DSA transport to GBM cells [22]. These approaches could help circumvent the limitations of BBB permeability, but further research is needed to determine whether seco-DSA itself can effectively reach GBM tumors.

To date, the cellular mechanisms responsible for the cytotoxicity of DSA and seco-DSA remain inadequately understood or studied. Our hypothesis is that seco-DSA’s extreme cytotoxicity could be an integral part of GBM and other tumors targeted by ADC therapy. To ascertain this hypothesis, we thoroughly characterized the cytotoxic effect as well as the mechanism of action of seco-DSA in GBM cell lines. At picomolar concentrations, seco-DSA demonstrated a concentration-dependent decrease in cell proliferation, colony formation, and metabolic activity. While treatment with seco-DSA-induced minimal apoptotic and necrotic cell death, it notably resulted in a significant arrest in the S and G2/M phases of the cell cycle and inhibited cell migration.

## 2. Results

### 2.1. Both DSA and Seco-DSA Show Significant Potency in GBM Cell Lines

**Figure 1:**
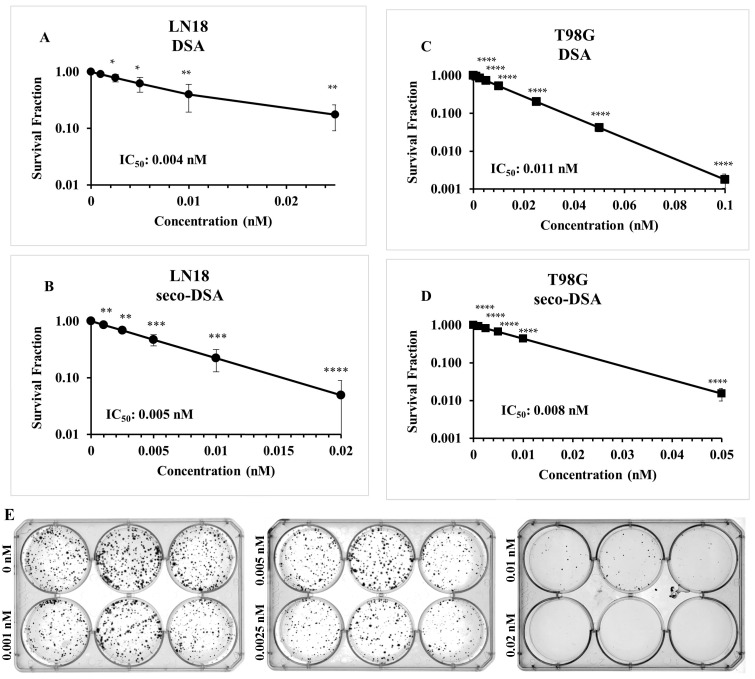
Both DSA and seco-DSA show significant potency in GBM cell lines. Both DSA and seco-DSA induce a concentration-dependent decrease in colony formation in T98G and LN18 cells, with their IC_50_ values being close and comparable within each cell line. Panels (**A**–**D**) show the IC_50_ values measured with the colony assay formation for each cell line used after treatment with DSA and seco-DSA. (**E**) Representative images of colony formation following seco-DSA treatment in LN18 cells. Data were normalized to control and are expressed as the average ± SD from three independent experiments. Statistical significance was assessed using the following criteria: * *p* < 0.05, ** *p* < 0.01, *** *p* < 0.001, **** *p* < 0.0001 compared to the control group, treated with (0.5% DMSO).

To establish the baseline response and assess the efficacy of DSA and seco-DSA, colony formation assays were conducted on LN18 and T98G cell lines. Cells were treated with varying concentrations of DSA and seco-DSA 24 h after seeding, and colony counts were performed once the control colonies exceeded 50 cells each. The IC_50_ for LN18 was determined to be 0.004 nM for DSA and 0.005 nM for seco-DSA (Figure 1A,B). In T98G cells, the IC_50_ values were 0.011 nM for DSA and 0.008 nM for seco-DSA (Figure 1C,D). These findings suggest that LN18 displays slightly greater sensitivity to both seco-DSA and DSA compared to T98G, with statistical analysis confirming that the IC_50_ value for LN18 was significantly lower than that of T98G (*p* < 0.05 for seco-DSA and *p* < 0.001 for DSA). Moreover, the results indicate that both DSA and seco-DSA exhibit significant potency in GBM cell lines. Based on these results, all subsequent experiments in this paper will use seco-DSA.

### 2.2. Seco-DSA Inhibits Cell Viability in GBM Cells

**Figure 2:**
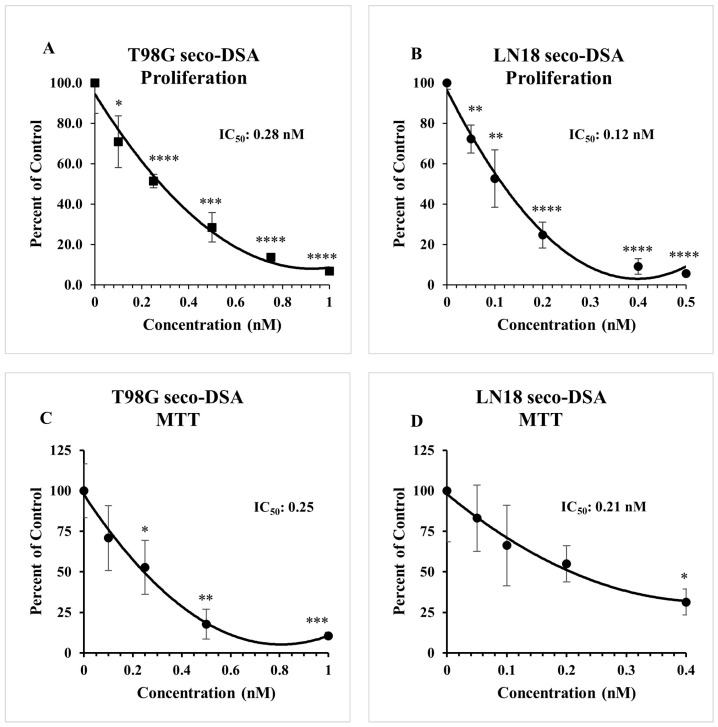
Seco-DSA inhibits cell viability in GBM cells. The cell proliferation assay was assessed using the trypan blue exclusion assay, and metabolic activity was assessed using an MTT assay. (**A**) The IC_50_ of seco-DSA for T98G cells in the cell proliferation assay is 0.28 nM. (**B**) The IC_50_ of seco-DSA for LN18 cells in cell proliferation assay is 0.12 nM. (**C**) The IC_50_ of seco-DSA for T98G cells in the MTT assay is 0.25 nM. (**D**) The IC_50_ of seco-DSA for LN18 cells in the MTT assay is 0.21 nM. Data were normalized to control and are expressed as the average ± SD from three independent experiments in triplicates. Statistical significance was assessed using the following criteria: * *p* < 0.05, ** *p* < 0.01, *** *p* < 0.001, **** *p* < 0.0001 compared to the control group.

Initial concentration-ranging studies were conducted to characterize the baseline response of LN18 and T98G cells to seco-DSA. Cell proliferation and MTT assays were then performed to assess cell viability and determine the IC_50_ values. The cells were treated with varying concentrations of seco-DSA 24 h after seeding and collected 72 h post-treatment. In the cell proliferation assay, the IC_50_ values were found to be 0.28 nM and 0.12 nM for T98G and LN18, respectively (Figure 2A,B). Similarly, for MTT assays, the IC_50_ values were determined to be 0.24 nM for T98G and 0.21 nM for LN18 (Figure 2C,D). These findings highlight the ultra-potency of seco-DSA in both LN18 and T98G cells, demonstrating its exceptional effectiveness in inhibiting proliferation in these chemo-resistant cell lines. Notably, LN18 exhibits greater sensitivity to seco-DSA compared to T98G in both assays, indicating potential cell-line-specific differences in response to seco-DSA. Additionally, the colony formation assay proved to be a more sensitive metric for evaluating seco-DSA’s potency, as it showed lower IC_50_ values compared to cell proliferation and MTT assays. This suggests that colony formation assay is a more stringent indicator of long-term cell survival following treatment (Figure 1 and Figure 2).

### 2.3. Seco-DSA Achieves Maximal Efficacy Within 8 h

**Figure 3:**
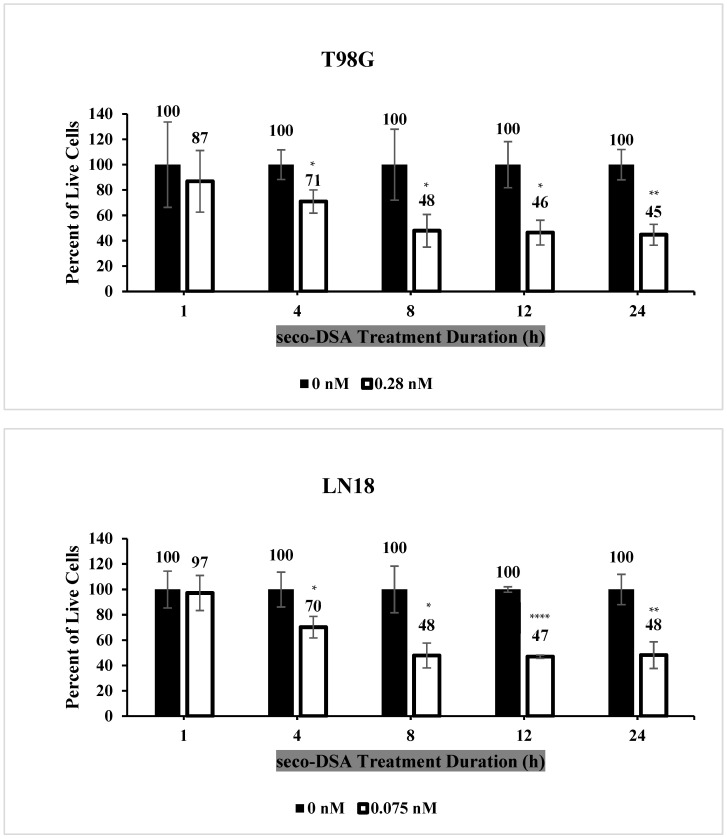
Seco-DSA achieves maximal efficacy within 8 h. T98G and LN18 cells were treated with seco-DSA at their respective IC_50_ concentrations for 1, 4, 8, 12, or 24 h. After each treatment, the media was replaced with fresh, drug-free media, and the cells were incubated for an additional 48 h. Live cells were then counted using the trypan blue exclusion assay. Black bars represent the control group treated with 0.5% DMSO (0 nM seco-DSA), while white bars represent the seco-DSA-treated group (0.28 nM for T98G and 0.075 nM for LN18). Each bar shows the mean percentage of live cells normalized to its own control. Treatment with seco-DSA led to a time-dependent decrease in cell viability, with significant reductions observed as early as 2 h, reaching a plateau at 8 h. Data represents the mean ± SD from three independent experiments performed in triplicate. Statistical significance was determined using the following criteria: * *p* < 0.05, ** *p* < 0.01, *** *p* < 0.001, **** *p* < 0.0001 compared to the control group.

To further characterize the kinetic profile of seco-DSA, which both needs to convert to DSA and then alkylate DNA to produce its cytotoxic effects, LN18 and T98G cell lines were treated with 0.28 nM and 0.075 nM of seco-DSA, respectively, and incubated for 1, 4, 8, 12, and 24 h. After each incubation period, culture medium was replaced with fresh medium, and cells were counted using the trypan blue exclusion assay 48 h after treatment (Figure 3). The data indicated that 1 and 4 h of seco-DSA incubation were insufficient for it to attain its full cytotoxic effect, although a statistically significant decrease in cell viability was observed at 4 h in both cell lines (*p* < 0.05). From 8 h onwards, seco-DSA reached a plateau, achieving the expected IC_50_, indicating that 8 h of incubation is sufficient for seco-DSA to reach its threshold. These findings suggest that seco-DSA achieves maximal efficacy within 8 h, making this threshold critical for future kinetic studies.

### 2.4. Extent of Apoptosis and Necrosis Does Not Fully Account for Significant Reduction Observed in Live Cells

**Figure 4:**
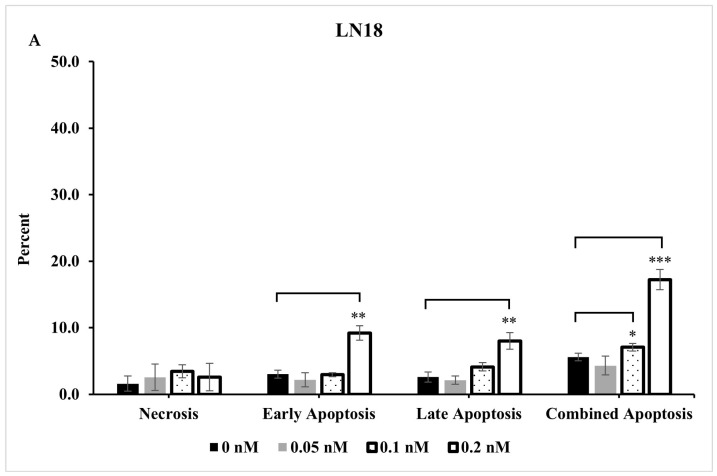

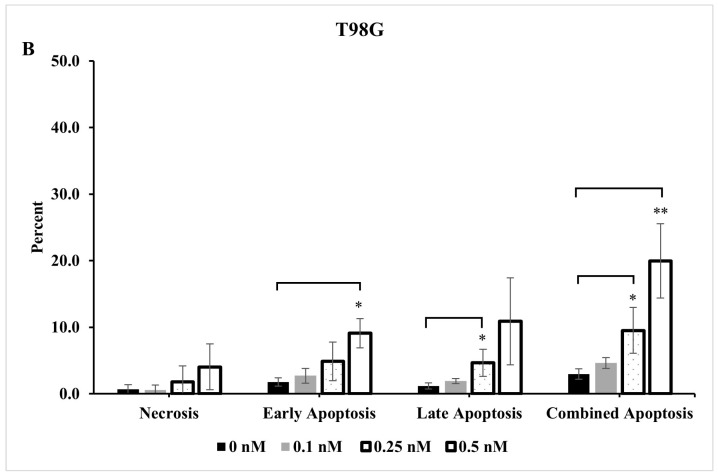
The extent of apoptosis and necrosis does not fully account for significant reduction observed in live cells. Effects of seco-DSA on early apoptosis, late apoptosis, combined apoptosis, and necrosis in LN18 and T98G cells evaluated 72 h post-incubation and analyzed by flow cytometry. The y-axis represents the percentage of cells undergoing each process, while the x-axis categorizes the type of cell death: necrosis (AV−/7AAD+), early apoptosis (AV+/7AAD−), late apoptosis (AV+/7AAD+), and combined apoptosis (AV+/7AAD− and AV+/7AAD+). (**A**) LN18 cells: the black bar represents control cells treated with 0.5% DMSO, the gray bar represents cells treated with 0.05 nM seco-DSA, the dotted bar represents cells treated with 0.1 nM seco-DSA, and the white bar represents cells treated with 0.2 nM seco-DSA. (**B**) T98G cells: the black bar represents control cells treated with 0.5% DMSO, the gray bar represents cells treated with 0.1 nM seco-DSA, the dotted bar represents cells treated with 0.25 nM seco-DSA, and the white bar represents cells treated with 0.5 nM seco-DSA. Seco-DSA induced a concentration-dependent increase in early, late, and combined apoptosis in both cell lines, although this increase did not fully account for the significant reduction in viability and metabolic activity observed. Minimal effects on necrosis were seen across all concentrations. Each bar represents the mean percentage of cells, with data expressed as the average ± SD from three independent experiments performed in triplicate. Statistical significance was determined using the following criteria: * *p* < 0.05, ** *p* < 0.01, *** *p* < 0.001, **** *p* < 0.0001 compared to the control group.

To investigate whether the decrease in live cell count resulted from apoptotic and/or necrotic cell death, LN18 and T98G cells were treated with various concentrations of seco-DSA, collected 72 h post-treatment, and stained with Annexin-V and 7AAD. Both cell lines exhibited a concentration-dependent increase in early, late, and total apoptosis in response to seco-DSA treatment (Figure 4). However, at the highest concentration of seco-DSA, the percentage of apoptotic cells was relatively low, with 17% of LN18 and 20% of T98G undergoing combined apoptosis. Additionally, necrosis was also minimal in both cell lines, with less than 5% of cells being necrotic. The results indicate that seco-DSA induces apoptotic cell death in LN18 and T98G cell lines, though the extent of apoptosis does not fully account for the significant reduction in live cell count observed in prior cell survival assays. Other mechanisms, such as cell cycle arrest or non-apoptotic forms of cell death, might contribute to the observed cytotoxicity.

### 2.5. Cell Cycle Progression Is Impacted by Seco-DSA

**Figure 5:**
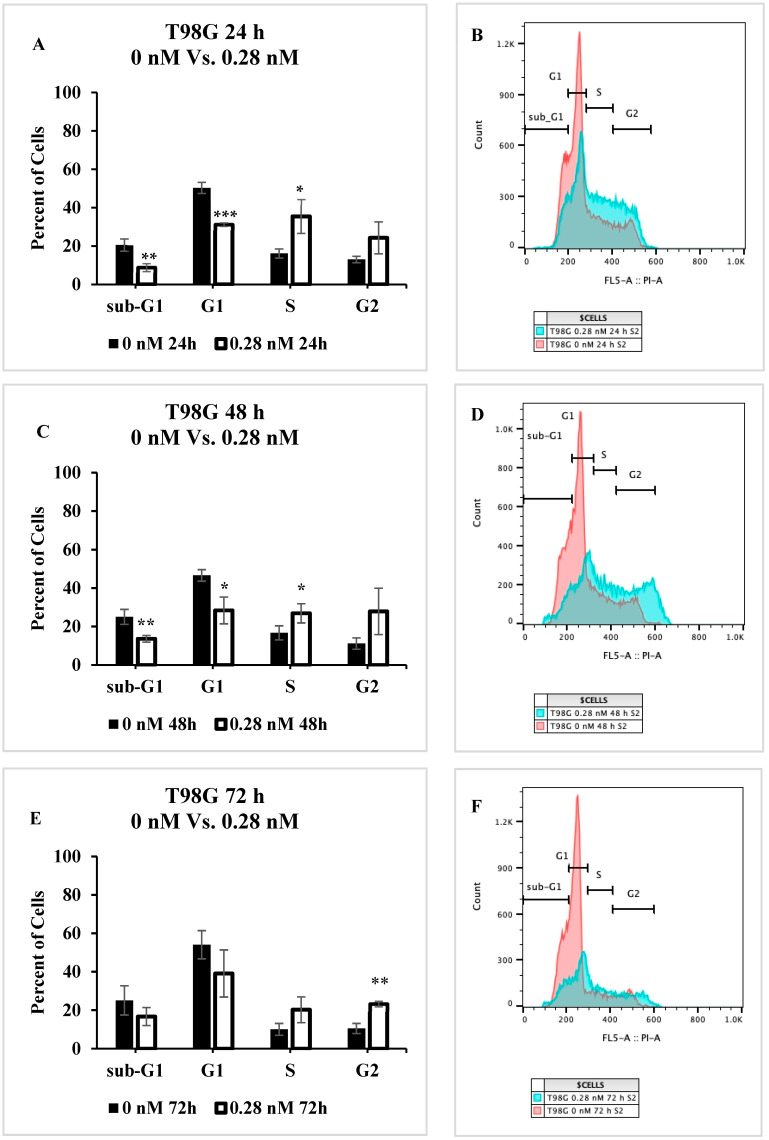

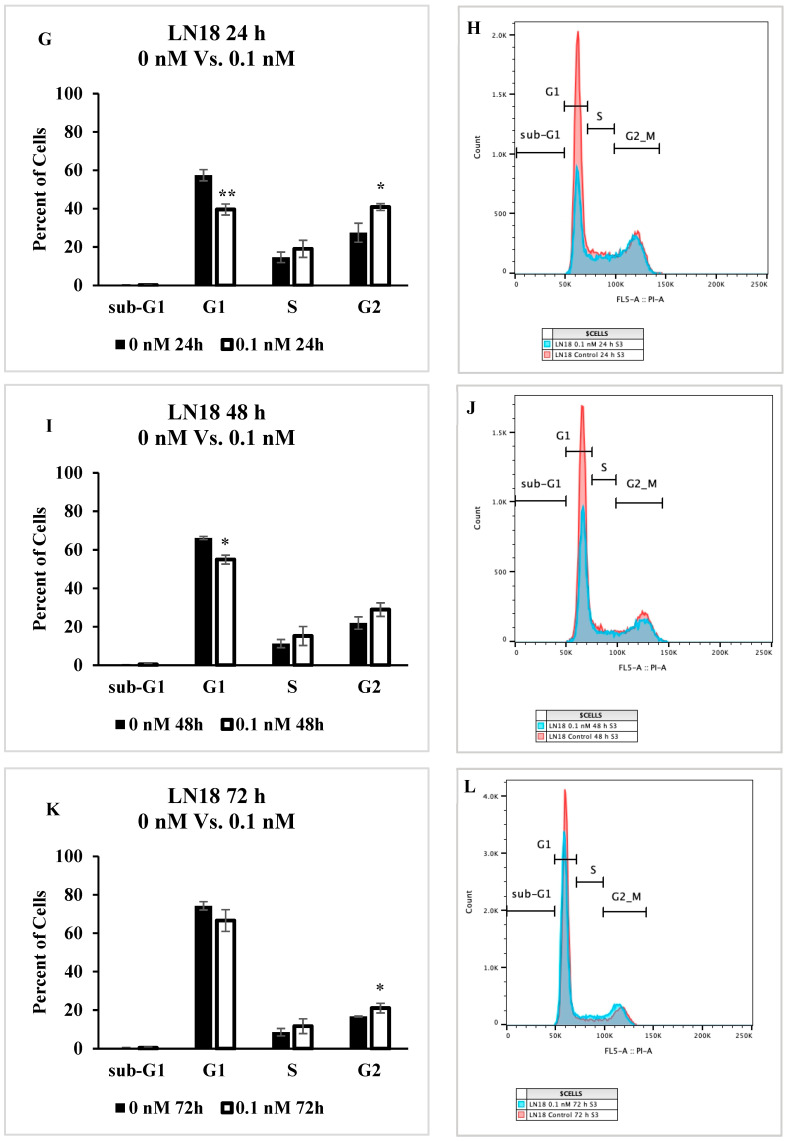
Cell Cycle Progression impact induced by seco-DSA. Seco-DSA was added 24 h after seeding, and cell cycle phases were analyzed at 24, 48, and 72 h post-treatment. The x-axis represents the different phases of the cell cycle determined using flow cytometry. The y-axis represents the percentage of cells in each phase. The black bars represent the control treated with 0.5% DMSO (0 nM seco-DSA), and the white bars represent the treated samples (0.28 nM for T98G and 0.1 nM for LN18). (**A**,**B**) T98G cells at 24 h post-treatment with 0 nM and 0.28 nM seco-DSA. (**C**,**D**) D54 cells at 48 h post-treatment with 0 nM and 0.28 nM seco-DSA. (**E**,**F**) T98G cells at 72 h post-treatment with 0 nM and 0.28 nM seco-DSA. (**G**,**H**) LN18 cells at 24 h post-treatment with 0 nM and 0.1 nM seco-DSA. (**I**,**J**) LN18 cells at 48 h post-treatment with 0 nM and 0.1 nM seco-DSA (**K**,**L**) LN18 cells at 72 h post-treatment with 0 nM and 0.1 nM seco-DSA. Each bar represents the mean percentage of cells. The data are expressed as the average ± SD from three independent experiments in triplicates. Statistical significance was assessed using the following criteria: * *p* < 0.05, ** *p* < 0.01, *** *p* < 0.001, **** *p* < 0.0001 compared to the control group.

Since the extent of apoptotic and necrotic cell death observed after seco-DSA treatment did not correlate to the observed proliferation arrest, we next examined whether seco-DSA induces a phase-specific cell cycle arrest in human GBM cells. T98G and LN18 cells were treated with 0.28 nM and 0.1 nM seco-DSA, respectively, 24 h post-seeding and subsequently stained with PI/RNase solution at 24, 48, and 72 h. At 24 h, cell cycle analysis revealed a significant decrease in the G1 phase (*p* < 0.001 for T98G and *p* < 0.01 for LN18), accompanied by an increase in the S and G2/M phases in T98G and a significant increase in the G2/M phase in LN18 (Figure 5A,G). At 48 and 72 h, the arrest continued in the S and G2/M phases in T98G and in the G2/M phase in LN18 (Figure 5C,E,I,K), though a larger cell population was arrested at the 24 h time point in both cell lines. Statistical analysis confirmed that the differences in cell cycle distribution were significant at each time point (*p* < 0.05). These findings strongly suggest that seco-DSA exerts a substantial arrest in both the S and G2/M phases of the cell cycle with a time-dependent shift toward G2/M phase arrest in both cell lines. The cell cycle arrest pattern likely contributes to the inhibition of proliferation observed in previous assays.

### 2.6. Cell Migration Is Inhibited by Seco-DSA

**Figure 6:**
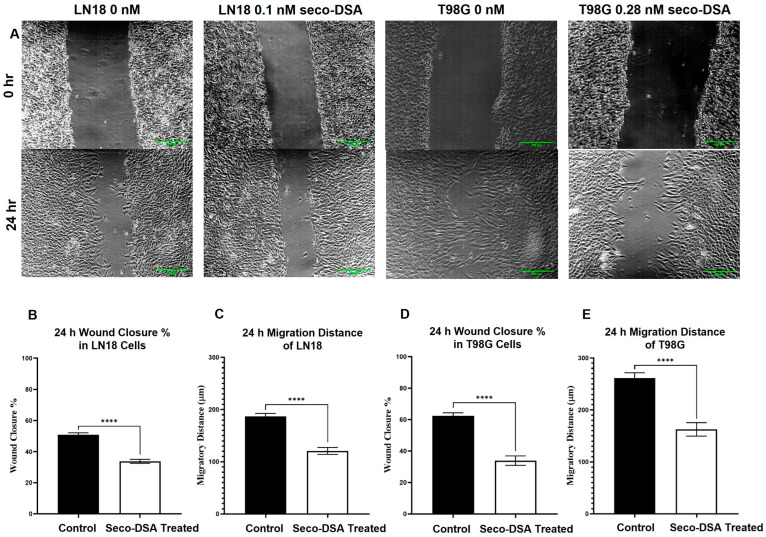
Seco-DSA inhibits migration in LN18 and T98G cells. Migration capacity was assessed using 24 h wound healing assays (n = 9). LN18 cells were treated with 0.1 nM seco-DSA, and T98G cells were treated with 0.28 nM seco-DSA. Control cells were treated with 0.5% DMSO treatment. (**A**) Representative images of wound healing at 0 h and 24 h (scale bar = 200 μm). (**B**,**D**) Wound closure percentages after 24 h: the LN18 control group achieved 51%, the LN18 seco-DSA-treated group achieved 34%, the T98G control group achieved 62%, and the T98G seco-DSA-treated group achieved 34%. (**C**,**E**) Migration distances after 24 h: LN18 Control group migrated 187 µm, LN18 seco-DSA-treated group migrated 121 µm, T98G Control group migrated 261 µm, and T98G seco-DSA-treated group migrated 162 µm. The data are expressed as the average ± SD from three independent experiments in triplicates. Statistical significance. * *p* < 0.05, ** *p* < 0.01, *** *p* < 0.001, **** *p* < 0.0001 compared to the control group.

In the scratch assay, DSA significantly reduced migration in both LN18 and T98G cells. On average, LN18 control samples exhibited a wound closure of 187 µm over 24 h, while DSA-treated samples had a closure of 121 µm. In terms of percentage, the LN18 control group achieved 51% wound closure, compared to 34% in the DSA-treated group. Similarly, T98G control samples showed an average closure of 261 µm, while DSA-treated samples had a closure of 162 µm. This corresponds to 62% wound closure in the control group, versus 34% in the DSA-treated group (Figure 6). This represents a 33% reduction in LN18 and a 45% reduction in T98G in the extent of wound closure in response to seco-DSA compared to the untreated group.

### 2.7. Proteomics Analysis

**Figure 7:**
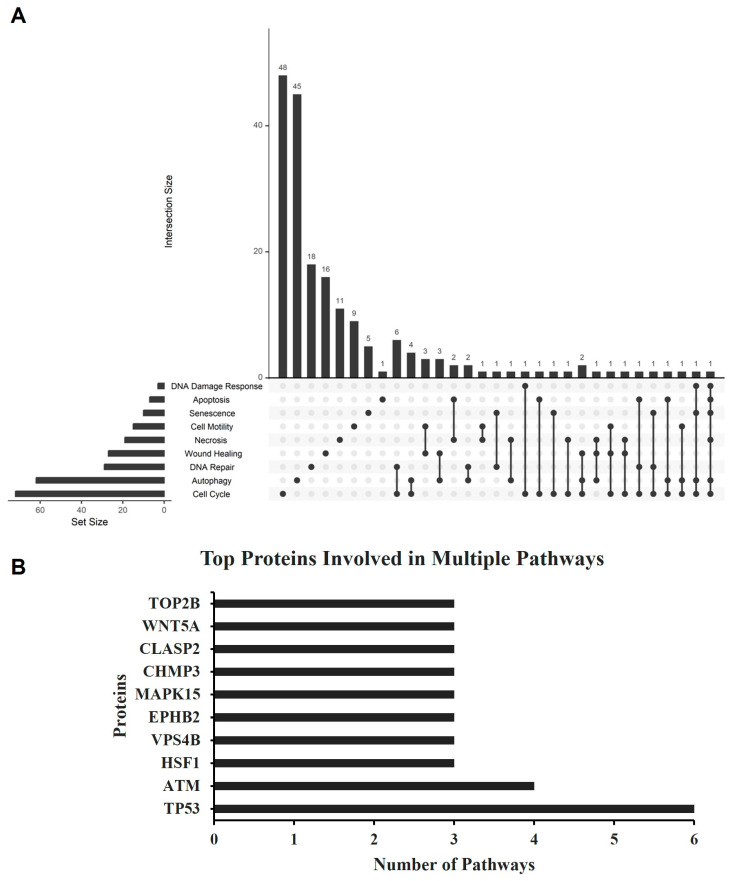
Cellular pathway intersections induced by seco-DSA treatment in LN18 cells. (**A**) The UpSet plot displays the overlap among pathways activated by seco-DSA treatment in LN18 cells, highlighting the number of proteins involved in each combination of pathways. The pathways analyzed include DNA damage response, apoptosis, senescence, cell motility, necrosis, wound healing, DNA repair, autophagy, and cell cycle. Bars represent the intersection size, indicating the number of proteins shared across specific pathway combinations. The set size bar on the left shows the total number of proteins associated with each pathway individually (LN18 Control, n = 1; LN18 DSA-treated, n = 1). (**B**) The bar chart presents the top proteins involved in multiple pathways activated by DSA treatment, with TP53 and ATM showing participation in the highest number of pathways (6 and 5, respectively), followed by HSF1, VPS4B, EPHB2, MAPK15, CHMP3, CLASP2, WNT5A, and TOP2B.

We initially investigated whether the proteomic profile of GBM cells treated with seco-DSA differed from non-treated GBM cells. Proteomic analysis was performed on LN18 cells, which were selected over T98G due to their higher sensitivity to seco-DSA. LN18 cells were treated with seco-DSA for 48 h, followed by protein isolation and proteomic analysis. Table 1 and Table 2 reveal distinct, differentially expressed proteins specific to cellular pathways triggered by seco-DSA treatment. These findings suggest that the cargo composition between each treatment status is sharply distinct, potentially serving as biomarkers for better understanding cell killing mechanisms of seco-DSA and resistance mechanisms of GBM. The UpSet plot (Figure 7A) shows the differential expression of proteins across various selected cellular processes, including DNA damage response, apoptosis, senescence, cell motility, necrosis, wound healing, DNA repair, autophagy, and cell cycle. The horizontal bars on the x-axis represent the total protein count for each cellular process, while the vertical bars on the y-axis represent the number of proteins distinct and shared between different combinations of processes.

In response to seco-DSA treatment, the most substantial differentially expressed proteins were unique to the cell cycle (48 proteins), followed by autophagy (45 proteins), suggesting that these cellular processes play a significant role in the cytotoxicity of seco-DSA. DNA repair and wound healing also showed a high number of differentially expressed proteins, with 18 and 16 proteins, respectively. Regarding cell death mechanisms, 11 proteins were differentially expressed in necrosis, and 5 were differentially expressed in senescence. Among apoptosis-related proteins, only the antiapoptotic BCL2L1 protein was found to be exclusive to apoptotic pathways and differentially expressed in the DSA-treated cells (Table 1). All other proteins associated with apoptosis that were differentially expressed compared to the control were not apoptosis-specific but shared with necrosis, cell cycle, DNA repair, autophagy, DNA damage, and senescence pathways.

The highest number of shared proteins is observed between DNA repair and the cell cycle, with six proteins common to both processes, followed by four proteins shared between the cell cycle and autophagy. Several minor intersections, each with only one shared protein, exist among other cellular process combinations, indicating a possible dual function role for these proteins. The bar graph (Figure 7B) illustrates the ten proteins involved in multiple cellular pathways, as identified in the UpSet plot (Figure 7A). Each protein is labeled with the number of pathways in which it participates, highlighting its multifunctional role across various cellular processes (Figure 7B). Notably, TP53 is the most prevalent protein, upregulated in six distinct pathways, including apoptosis, necrosis, senescence, autophagy, the cell cycle, and DNA damage response (Table 1 and Table 2). ATM, on the other hand, is downregulated and shared among four pathways: senescence, autophagy, the cell cycle, and DNA damage response (Table 1 and Table 2).

Comprehensive data on the up- and down-regulated proteins across all pathways represented in the UpSet plot are detailed in Table 1 and Table 2. These tables provide an overview of the differential protein expression in response to seco-DSA in each pathway, including apoptosis, necrosis, senescence, autophagy, cell cycle, DNA damage, repair response, wound healing, and cell motility. The data highlight key regulatory proteins unique to or shared among these pathways, offering insights into the complex interplay of cellular mechanisms in response to seco-DSA treatment at 48 h.

## 3. Discussion

The duocarmycin class of antitumor antibiotics represents an exceptionally potent group of agents that exert their biological effects through sequence-selective alkylation of duplex DNA [12]. GBM cells are known for their remarkable resistance to alkylating drugs. Specifically, GBM cell lines such as LN18 and T98G overexpress elevated levels of O6-methylguanine-DNA methyltransferase (MGMT) and exhibit significant resistance to current FDA-approved alkylating agent, such as temozolomide [23,24]. TMZ, an alkylating agent, methylates guanine at the N7 position [25]. Glioblastoma cell lines expressing elevated levels of MGMT, such as LN18 and T98G, exhibit resistance to TMZ [23,24]. In colony formation assays, the IC_50_ of TMZ exceeds 500,000 nM in LN18 and T98G cells [24], compared to just 0.005 nM and 0.008 nM for seco-DSA, respectively, highlighting seco-DSA’s superior potency. Interestingly, seco-DSA and its parent compound, DSA, exhibit minimal differences in IC_50_ values, with seco-DSA at 0.005 nM in LN18 and 0.008 nM in T98G, and DSA at 0.004 nM in LN18 and 0.011 nM in T98G. Structurally, seco-DSA differs from DSA by the removal of the cyclopropane ring and by the inclusion of a phenol group and a chlorine atom in the DNA-binding domain of the drug. Upon undergoing in situ spirocyclization, seco-DSA generates the cyclopropane ring characteristic of DSA [26]. The minimal differences in IC_50_ values between DSA and seco-DSA in both LN18 and T98G cells suggest that seco-DSA quickly converts to DSA once in the media and that DSA then interacts with the cells.

The preliminary data presented in this study is essential for understanding the initial response and sensitivity of resistant GBM cell lines to seco-DSA. The findings indicate that seco-DSA, like DSA, significantly reduces cell survival and inhibits metabolic activity at picomolar concentrations in both LN18 and T98G cells. Sustaining proliferative signaling is a hallmark of cancer, and seco-DSA’s ability to inhibit uncontrolled proliferation is critical for cancer therapy. This inhibition not only has the potential to slow tumor growth, making surgical resection more feasible, but also reduces the likelihood of metastasis, as shown by the results of colony formation, proliferation, and migration assays. In particular, in the scratch assay, seco-DSA performed better than the control by inhibiting the migration rate at 24 h. Proteomics analysis showed significant changes in the expression of several proteins associated with wound healing. These findings indicate seco-DSA’s strong anti-migratory effect, which may play a key role in limiting the spread of GBM cells and contributing to its therapeutic potential in managing tumor progression, tumor invasion, and extending survival.

Our study revealed that the IC_50_ value derived from the colony formation assay was lower than that obtained from the MTT assay. This underscored fundamental differences in what these assays measure. The MTT assay primarily assesses cell viability by measuring metabolic activity, relying on mitochondrial enzymes to convert tetrazolium salts into formazan [27]. While useful for evaluating short-term cytotoxic effects, it does not account for long-term survival or proliferative capacity. In contrast, the colony formation assay measures long-term clonogenic survival by assessing the ability of single cells to proliferate into colonies. This method provides insight into the reproductive capacity of cells after exposure to treatment. The distinction between these assays has been recognized by other studies, but our findings emphasize its critical relevance in evaluating seco-DSA [28]. Our results suggest that seco-DSA may exert long-term cytostatic effects rather than immediate cytotoxicity, making the colony formation assay a more sensitive measure of its efficacy.

Supporting this, seco-DSA was found to induce minimal apoptosis and necrosis, significantly less than the decrease in cell survival observed in proliferation and MTT assays at the IC_50_. The observed discrepancy likely reflects differences in the mechanisms measured by these assays. The trypan blue assay captures an early loss of viability due to a broader range of cellular states, including senescence and arrested growth, while Annexin V/7-AAD specifically detects apoptotic or necrotic cells. This suggests that seco-DSA induces a cytostatic effect, rather than classical apoptotic or necrotic cell death. Seco-DSA might induce a non-apoptotic, non-necrotic form of growth inhibition, which would reduce the number of live cells in trypan blue counts but not register as apoptotic or necrotic in Annexin V/7-AAD staining. Supporting this observation, previously published work showed that treatment with 0.1 nM DSA as a stand-alone treatment in the U-138 GBM cell line induces minimal necrosis, suggesting that DSA alone has little impact on necrotic cell death [14].

Notably, seco-DSA induces a significant arrest in both the S and G2/M phases in T98G cells, while in LN18 cells it predominantly causes arrest in the G2/M phase, with the most significant phase arrest observed at the 24 h time point in both cell lines. The ability of seco-DSA to cause G2/M phase arrest highlights its potential as a radiosensitizer, given that the G2/M phase is the most sensitive to radiation [29]. This observation suggests the need for future studies exploring its synergistic effects in combination with radiation therapy, work that is ongoing in our laboratory. Proteomic analysis also revealed significant changes in 48 cell cycle proteins, suggesting that this pathway plays a pivotal role in mediating LN18’s response to seco-DSA. While this dataset defines the fundamental biological response of seco-DSA in human GBM cells, further investigations are essential to elucidate the underlying mechanisms behind its potency.

To explore the diverse cell death mechanisms and protein expressions activated by seco-DSA in LN18 cells, we utilized proteomics to gain deeper understanding into the pathways and key players involved. LN18 cells were selected for initial proteomic analysis due to their increased sensitivity to seco-DSA relative to T98G, offering insights into cellular responses that may be less observable in less sensitive cell lines. Though apoptosis is generally associated with alkylating agents [7], LN18 cells treated with seco-DSA exhibited minimal apoptosis-specific protein expression changes, with only one upregulated protein, BCL2L1, indicating an anti-apoptotic response [30]. The upregulation of BCL2L1 and the absence of differentially expressed caspase family members suggest LN18 cells are able to evade apoptotic cell death in the conditions tested. This finding complements the minimal apoptosis observed in response to seco-DSA when LN18 cells were stained with Annexin V (Figure 3). The remaining proteins involved in apoptosis that were shared across other cellular pathways like necrosis, cell cycle, DNA repair, autophagy, DNA damage, and senescence highlight the complex interplay between these processes in response to seco-DSA.

The identification of 18 differentially expressed proteins unique to DNA repair may indicate seco-DSA’s role in inducing DNA damage, which activates repair mechanisms to preserve genomic integrity. ATM, a key checkpoint protein, was dysregulated in seco-DSA-treated cells, potentially disrupting ATM-mediated repair mechanisms, thereby promoting the accumulation of DNA damage, triggering alternative cell death pathways, and alluding to similar mechanisms in radiation-induced DNA damage response. However, further experiments are needed to confirm ATM downregulation and its associated pathway proteins to elucidate their roles in the response to seco-DSA.

The discovery that seco-DSA induces the differential expression of 45 proteins specific to autophagy is both intriguing and novel, marking the first reported association between seco-DSA and autophagic mechanisms. The mixed pattern of up- and down-regulation among these autophagy-related proteins points toward autophagic dysfunction. For instance, the ATG12-ATG5-ATG16L1 complex is critical for autophagosome formation, while ATG2A is essential for lipid transfer during autophagosome membrane expansion [31,32,33]. Under seco-DSA treatment, ATG16L1 and ATG2A are upregulated, yet ATG5 is downregulated, suggesting that the formation of a functional ATG12-ATG5-ATG16L1 complex may be compromised. This imbalance implies that while autophagy is being initiated, the process may stall due to incomplete autophagosome assembly, leading to dysfunctional autophagy.

The disrupted autophagic process could have significant cellular consequences. When autophagy fails to proceed to completion, damaged organelles and protein aggregates may accumulate, increasing cellular stress and potentially triggering alternative stress responses or cell death pathways. This impaired autophagic flux may thus exacerbate cellular stress and could render cells more susceptible to additional cytotoxic effects of seco-DSA. Future investigations exploring the downstream impacts of these autophagy-related disruptions, particularly regarding cellular stress signaling pathways, could further elucidate the mechanistic role of seco-DSA in modulating autophagy.

In conclusion, this study represents a crucial first step in uncovering the mechanism of action of seco-DSA in human GBM cells, revealing its complex modulation of cellular pathways such as autophagy, DNA repair, cell cycle regulation, and cell migration. Our proteomic analysis identifies differential protein expression patterns, suggesting a multifaceted cellular response to seco-DSA. However, these preliminary insights require validation through targeted techniques, such as Western blotting, to confirm the up- and down-regulation of key proteins identified in each pathway, which will be critical for solidifying the proposed mechanistic hypotheses.

To deepen these findings, future research should employ more refined proteomic analyses, including the examination of post-translational modifications (PTMs) like phosphorylation, ubiquitination, and acetylation. Phosphorylation-specific proteomics could reveal critical regulatory points in signaling pathways, such as those involving ATM in the DNA repair and cell cycle pathways, which may be modulated by seco-DSA. These insights could pinpoint precise molecular targets of seco-DSA and provide a clearer picture of its impact on pathway dynamics and cellular stress responses. Together, these advanced analyses would expand our understanding of seco-DSA’s role in GBM treatment and potentially support its development as a targeted therapeutic agent.

## 4. Material and Methods

### 4.1. Cell Lines and Cell Culture

Human T98G (ATCC CRL-1690) and LN18 (ATCC CRL-2610) cell line cells were cultured in EMEM cell culture medium (Genesee Scientific, El Cajon, CA, USA) containing 10% fetal bovine serum (FBS, Genesee Scientific, El Cajon, CA, USA) and DMEM/high glucose cell culture medium (Cytiva, Marlborough, MA, USA) containing 5% FBS (Genesee Scientific, El Cajon, CA, USA), respectively. All cells were maintained at 37 °C and 5% CO_2_.

### 4.2. DSA Treatment

Stock solutions of seco-DSA were obtained by dilution in dimethyl sulfoxide (DMSO) to a final concentration of 100,000 nM and stored at −80 °C. Stock solutions of seco-DSA were diluted in cell culture media using a range of concentration between 0.0001 nM and 1 nM and added to cells prior to each experiment. Control cells were treated with 0.5% DMSO.

### 4.3. Colony Formation Assay

Cells were cultured in 6-well plates and incubated for two weeks at 37 °C and 5% CO_2_. After incubation, cells were fixed with ice-cold methanol and acetic acid (3:1) for 15 min and stained with 0.5% crystal violet for 2 h. Colonies, defined to consist of at least 50 cells, were scored manually. Data were fitted to single-target model using the equations SF = *e*(−D/D_0_). A single-target model is employed to define the IC_50_ value of seco-DSA and DSA, where IC_50_ is the dose/concentration that inhibits cell proliferation by 50%. In this equation, D is the delivered dose/concentration, and the D_0_ is the dose/concentration that inhibits proliferation by 63% [34].

### 4.4. Cell Proliferation Assay

Cells were seeded in triplicates in 6-well plates 24 h pre-treatment and incubated at 37 °C and 5% CO_2_. After detachment using trypan blue 0.25% (Genesee Scientific, El Cajon, CA, USA), cells were stained with trypan blue, 0.4% solution, and counted with an automatic cell counter, Countess II (Hyland Scientific, Stanwood, WA, USA). Data were fitted to a polynomial using the following equation: *y* = *ax*^2^ + *b* + *c*. The cell proliferation IC_50_ was calculated using the quadratic formula: $x=\frac{-b\pm \sqrt{b^{2}-(4a\left(c-50\right))}}{2a}$. The quadratic formula was employed to calculate the IC_50_ for this endpoint because it is a straightforward method that can directly measure the rate of cell division and growth.

### 4.5. Methyl Thiazolyl Tetrazolium (MTT) Assay

MTT powder (EMD Millipore Corp., Burlington, MA, USA) was dissolved in PBS (HyClone, Logan, UT, USA) at a concentration of 25 mg/mL, sterilized through a 0.22 μm filter. and stored in the dark at 4 °C until use. Cells were cultured as above in a 96-well microplate at a seeding density of 5000 cells per well. Cells were left to attach overnight and then treated with either 0.05, 0.1, 0.2, or 0.4 nM seco-DSA for LN18 and 0.1, 0.25, 0.5, or 1 nM seco-DSA for T98G. Control cells were treated with 0.5% DMSO. After 72 h of treatment, culture medium was removed, and cells were washed with PBS. Fresh culture medium (100 μL) was added to each well of the microplate along with 50 μL of MTT solution. The microplate was then incubated at 37 °C and 5% CO_2_ for 3 h. Plates were next spun at 500× *g* for 5 min, and medium was carefully removed from each well in order to not disturb the MTT formazan crystals formed at the bottom of each well. 100 μL of DMSO was added to each well, and the microplate was gently agitated on a shaker for 15 min to dissolve the MTT formazan crystals, after which the absorbance of the samples was read at 570 nm using a plate reader with a reference wavelength of 630 nm. The absorbance is proportional to the number of viable cells in each well.

### 4.6. Apoptosis and Necrosis Assay

Cells were seeded 24 h prior to seco-DSA treatment and collected at 72 h post-treatment. Triplicates of each treatment were prepared in a 96-well plate, and 150,000 cells were placed in each well. Cells were double stained using the Pacific Blue^TM^ Annexin V Apoptosis Detection Kit with 7-AAD (Biolegend, San Diego, CA, USA) and quantified using flow cytometry with a MACSQuant^®^ Analyzer 10 cytometer (Miltenyi Biotec, San Diego, CA, USA). Apoptotic and necrotic cells were analyzed using FlowJoTM Software for Mac Version 10.10.0 (BD Life Sciences, Ashland, OR, USA).

### 4.7. Cell Cycle Analysis

Control and seco-DSA-treated cells were collected 24, 48, and 72 h post-treatment, washed with PBS, and fixed with 70% ice-cold ethanol for >1 h at −20 °C. Cells were stained using FxCycle^TM^ PI/RNase staining solution and quantified using flow cytometry with a MACSQuant^®^ Analyzer 10 cytometer (Miltenyi Biotec, San Diego, CA, USA). Data were analyzed using FlowJoTM Software for Mac Version 10.10.0 (BD Life Sciences, Ashland, OR, USA).

### 4.8. Scratch Assay

Cells were seeded in wells according to their growth rates and phenotypes to achieve confluence overnight. LN18 cells were seeded at 6.0 × 10^5^ cells per well, and T98G cells at 5.5 × 10^5^ cells per well, in 12-well plates. Once cell culture reached confluence, the cell culture medium was removed and replaced with FBS-free medium, and the cells were subjected to serum starvation for 24 h. Scratches were made using a 10 μL pipette tip, followed by treatment with DSA at the IC50 concentration specific to each cell line. The scratches were monitored, and images were captured at set intervals using a Fisher Scientific Inverted Microscope (11350119) (Thermo Fisher Scientific, Waltham, MA, USA). Image analysis was performed using software (v1.53k, National Institutes of Health, Bethesda, MD, USA) [35].

### 4.9. Proteomic Analysis Sample Preparation

Cells were seeded in T-75 flasks, treated with seco-DSA, and the protein isolate was collected in a pellet. Samples were denatured with urea and reduced with DTT at 37 °C, followed by an IAA alkylation. The buffer was exchanged to ABC, and trypsin was added for a >16 h incubation. A Speed-Vac was used for peptide concentration, then they were desalted using C18 Zip Tip. Peptides were dried using a Speed-Vac and stored until LC-MS/MS analysis. Sample preparation and proteomics analysis were performed at the Proteomics Core Facility IIGB—University of California, Riverside.

### 4.10. LC-MS/MS Data Acquisition

The fractions were resuspended with 20 μL of water with 0.1% formic acid, separated by nano-LC, and analyzed by on-line electrospray tandem mass spectrometry. The experiments were performed on an EASY-nLC 1200 system (Thermo Fisher Scientific, Waltham, MA, USA) connected to a quadrupole-Orbitrap mass spectrometer Orbitrap Fusion Tribrid Mass Spectrometry equipped with an EASY-Spray ion source. A 5 μL peptide sample was loaded onto the trap column (Thermo Fisher Scientific Acclaim PepMap C18, 75 μm × 2 cm) with a flow of 10 μL/min for 3 min and subsequently separated on the analytical column (Acclaim PepMap C18, 75 μm × 25 cm) with a linear gradien, from 3% D to 37% D in 180 min. The column was re-equilibrated at initial conditions for 5 min. The flow rate was maintained at 300 nL/min, and the column temperature was maintained at 45 °C. The electrospray voltage of 2.2 kV versus the inlet of the mass spectrometer was used. The Orbitrap Fusion Mass Spectrometry was operated in the data-dependent mode to switch automatically between MS and MS/MS acquisition. Survey full-scan MS spectra (*m*/*z* 375–1500) were acquired with a mass resolution of 60 K, followed by fifteen sequential high energy collisional dissociation (HCD). The AGC target was set to 400,000, and the maximum injection time was 100 ms. MS/MS acquisition was performed in the ion trap. The AGC target was set to 3000, and the isolation window was 1.6 *m*/*z*. Ions with charge states 2+, 3+, and 4+ were sequentially fragmented by higher energy collisional dissociation (HCD) with a normalized collision energy (NCE) of 35%; a fixed first mass was set at 100. In all cases, one micro scan was recorded using dynamic exclusion of 30 s. The raw data were processed and analyzed using MaxQuant (version 2.1.4.0) with a homemade database. Mass tolerances for precursor and fragment ions were 6 and 10 ppm, respectively, the minimum peptide length was 6 amino acids, and the maximum number of missed cleavages for trypsin was 2.

### 4.11. LC-MS/MS Data Search

The raw data were processed and analyzed using MaxQuant (version 2.1.4.0) with a homemade database. Mass tolerances for precursor and fragment ions were 6 and 10 ppm, respectively, the minimum peptide length was 6 amino acids, and the maximum number of missed cleavages for trypsin was 2.

### 4.12. Protein Mass Spec Analysis

Peak-intensity data collected from the Proteomics Core Facility IIGB—University of California, Riverside were used for differential expression between control and 0.1 nM DSA-treated groups. Log-Fold Change (LFC) values were collected relative to control peak intensity. A baseline threshold of 2 LFC was chosen to qualify proteins for further analysis. Zero values were abbreviated to presence-absence within trial data, relative to control, and were included in the final analysis. The package clusterProfiler [36] was used in R version 4.4.1 [37] to obtain biological processes ontology annotations for differentially expressed proteins. Annotation reports were filtered for proteins involved in selected pathways. All proteins found differentially expressed and present in selected pathways were assigned arrows indicating a change in expression relative to control. Upset plots were created using UpSetR for data visualization [38].

### 4.13. Mass Spec Proteomics Data Accessibility

The mass spectrometry proteomics data have been deposited to the ProteomeXchange Consortium (https://proteomecentral.proteomexchange.org, accessed on 27 February 2025) via the PRIDE partner repository with the dataset identifier PXD061023 [39].

### 4.14. Statistical Analysis

All experiments, with the exception of proteomics data, were performed in biological and technical triplicates. The two-tailed unpaired *t*-test was used to determine statistically significant differences, and statistical significance was determined at *p* value ≤ 0.05.

## Figures and Tables

**Table 1:** Differential expression of cell death-related proteins in response to seco-DSA treatment, indicating upregulation and downregulation patterns.

	Apoptosis	Necrosis	Senescence	Autophagy		
**Up**	TP53	TP53	TP53	TP53	ATG16L1	IKBKG
	BCL2L1	AKAP12	TOP2B	MET	ATP6V0A1	EHMT2
		ASAH1	MME	RAB5A	VTI1A	WNK1
		TRADD	MAP2K4	TRIM23	SCOC	MAPK15
		INPP5K		ATG2A	EXOC7	EPHB2
		EPHB2		WDR45B	MTCL1	
		YTHDC2		ACBD5	ATP6V1D	
**Down**	HTRA2	CASP4	ATM	HTRA2	CALCOCO2	RNF5
	CDK5RAP3	FADD	SMC5	VPS37C	FKBP8	FYCO1
	HSF1	GBA1	HMGA2	LGALS8	WDR81	MLST8
	CASP4	WNT5A	ERCC1	HGS	RNF213	ATG5
	FADD	CHUK	HMGA1	SPTLC2	MARK2	SH3BP4
		BCL10	ZNF277	ATP6V1C1	WDFY3	WDR45
		GBP2		ATP6V1E1	MTMR14	TRIM21
		TMSB4X		ARFIP2	PIK3C3	CDK5
		CD47		RAB8A	SVIP	ATM
		SMPD4		UFM1	PIP4K2C	VPS4B
		NUB1		FOXK1	RMC1	CHMP3
		HDAC2		BNIP3	SNF8	SETD2
				FUNDC2	SPG11	TIGAR
				CHMP4A	SNX18	GBA1
				CHMP1B		

**Table 2:** Differential expression of cell cycle, DNA damage and repair, cell motility, and wound healing related proteins in response to seco-DSA treatment, indicating upregulation and downregulation patterns.

	Cell Cycle		DNA Damage	DNA Repair	Cell Motility	Wound Healing
**Up**	TP53	PDGFRB	TP53	POLE	MAPK15	EPHB2
	EHMT2	CCNA2	NBN	SPIRE1	WASL	RAB27A
	WNK1	CCNH		DTX3L	SYNJ2BP	VAV2
	MAPK15	PLK1		TOP2B	STK26	FERMT3
	CIT	RAD23A		HDAC3	STARD13	RAP2B
	XRCC3	KIF14		POLD3	RAP2B	
	SYF2	KANK2		SUPT20H		
	POLE	DTX3L		WRAP53		
	SPIRE1	TOP2B		CBX8		
	TOM1L2	LCMT1				
	CENPS	NBN				
	ANAPC13	YTHDC2				
	TAOK3	FAM83D				
**Down**	HTRA2	MCM4	ATM	HSF1	CLASP2	VPS4B
	CDK5RAP3	MLH1		SETD2	ADCY3	CHMP3
	HSF1	CDC34		TIGAR	NEURL1	CHMP1B
	TRIM21	UBE2A		RAD51	ENG	FUNDC2
	CDK5	CKS1B		SMARCC2	RABGEF1	CHMP4A
	ATM	SRPK2		BABAM2	ZMYND8	WNT5A
	VPS4B	CDC20		TADA3	HDAC2	CLASP2
	CHMP3	DLG1		SUPT7L	GNA13	PAPSS2
	KIF3B	IK		NSD2	MIA3	GPX1
	ANKRD17	TOP3A		YY1		ITGA5
	PIAS1	FOSL1		POLE2		YAP1
	RAD51	CLASP2		MORF4L2		METAP1
	SMARCC2	CUL3		APLF		HPS6
	BABAM2	GINS1		MCRS1		AJUBA
	WNT5A	CDC73		PNKP		VKORC1
	SMC5	MYO16		SENP3		ARHGAP35
	UBE2E2	GSPT1		HPF1		AK3
	TUBGCP2	APPL2		PPP4R2		SLC7A11
	RIOK2	TK1		TERF2IP		HPSE
	TIPIN	SMC2		HMGA2		F11R
	NLE1	GIGYF2				GNA13
	C9orf78	CTDP1				MIA3
	TELO2	MAD1L1				

## Data Availability

The raw data supporting the conclusions of this article will be made available by the authors on request. Proteomics data are available via ProteomeXchange with identifier PXD061023.
